# Inhibitory Effects of Bisphenol Z on 11β-Hydroxysteroid Dehydrogenase 1 and In Silico Molecular Docking Analysis

**DOI:** 10.3390/molecules30193941

**Published:** 2025-10-01

**Authors:** Tomasz Tuzimski, Mateusz Sugajski

**Affiliations:** 1 Department of Physical Chemistry, Faculty of Pharmacy, Medical University of Lublin, Chodźki 4a, 20-093 Lublin, Poland; 2 Department of Environmental Chemistry and Bioanalytics, Faculty of Chemistry, Nicolaus Copernicus University, Gagarina 7, 87-100 Torun, Poland; mateusz.sugajski@o2.pl

**Keywords:** bisphenol Z (BPZ), kinetic analysis, 11β-hydroxysteroid dehydrogenase 1 (11β-HSD1), corticosterone (CORT), Lineweaver-Burk plots, Eadie-Hofstee plots, Hanes-Woolf plots, molecular docking

## Abstract

Bisphenol A (BPA) is classified as an endocrine disruptor that mainly mimics the effects of estrogen and disrupts the synthesis of male androgens. Due to the toxicity of BPA, some new analogs, such as bisphenol BPB, BPC, BPF, PBH, and BPZ, were introduced into the market. The goal of this research was to demonstrate the applicability of kinetic analysis, in particular, Lineweaver-Burk plots, in assessing the impact of bisphenol Z on enzymatic activity. This study aimed to characterize the inhibitory effects of BPZ on 11β-hydroxysteroid dehydrogenase 1 (11β-HSD1) activity in the transformation of 11-dehydrocorticosterone (DHC) to corticosterone (CORT). During the determination of the enzymatic reaction product, chromatographic analysis conditions were optimized using gradient elution and an Acquity UPLC BEH C18 chromatographic column. The retention time of the assayed corticosterone was approximately 2 min. Also described and compared were graphical methods of analysis and data interpretation, such as Lineweaver-Burk, Eadie-Hofstee, and Hanes-Woolf plots. The experiments demonstrated that bisphenol Z is a mixed 11β-hydroxysteroid dehydrogenase 1 (11β-HSD1) inhibitor, responsible for catalyzing the conversion of 11-dehydrocorticosterone (DHC) to corticosterone (CORT). This relationship was confirmed by analyzing Lineweaver-Burk plots, which showed an increase in apparent *K_M_* with a decrease in the constant *V_max_*, suggesting a mixed inhibition mechanism. Molecular docking and detailed analysis of the interaction profiles revealed that BPZ consistently occupies the active site cavities of all examined enzymes (rat and human 11β-HSD1 and Arabidopsis 11β-HSD2), forming a stabilizing network of non-covalent interactions. Our research has significant biological significance considering the role of the 11β-HSD1 enzyme in the conversion of DHC to CORT and the importance of this process and its functions in adipose tissue, the liver, and the brain.

## 1. Introduction

**Figure 1:**
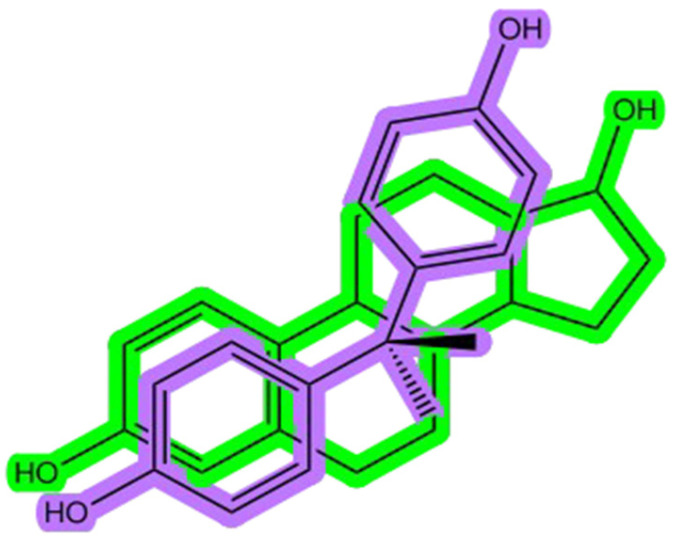
The bisphenol A (BPA) structure (shown in purple) overlapped with the estradiol chemical structure (shown in green).

Bisphenols, such as bisphenol A (BPA), are synthetic organic compounds widely used in industry (e.g., the production of plastics and resins), which have garnered significant scientific concern due to their endocrine-disrupting properties. Endocrine-disrupting chemicals (EDCs) are substances capable of altering hormones or interacting with the endocrine system in organisms. Bisphenols’ endocrine-disrupting properties and severe toxic effects have led to widespread international restrictions on BPA and the production and use of some analogs worldwide. Structurally, BPA is notably similar to 17β-estradiol, the primary endogenous estrogen hormone (Figure 1). The structural resemblance of the two phenolic rings, which facilitates hydrogen bonding and π-π interactions, underpins their ability to bind to estrogen receptors (ERα and ERβ), mimicking natural estrogenic activity [1]. BPA and related analogs can act not only as weak agonists but also as partial antagonists, depending on the receptor subtype and concentration, thereby altering normal endocrine signaling pathways in a manner similar to selective estrogen receptor modulators (SERMs) [2].

Estrogen-related receptors, namely, ERRα, ERRβ, and ERRγ (also known as ERR1–3), are another subfamily of orphan nuclear receptors with sequence similarity to ESR1 [3]. Bisphenol A (BPA) and its analogs can bind both estrogen receptors (ESR1 and ESR2) and estrogen-related receptors (ERR1–3), which block Leydig cell gene expression and bind to the androgen receptor (NR3C4) as an antagonist to block the activation of Leydig cell genes [3]. BPA can also bind membrane G-coupled receptors (GPER) or ESR1, which activate the ERK1/2 pathway to inhibit the differentiation of Leydig cells [3].

**Figure 2:**
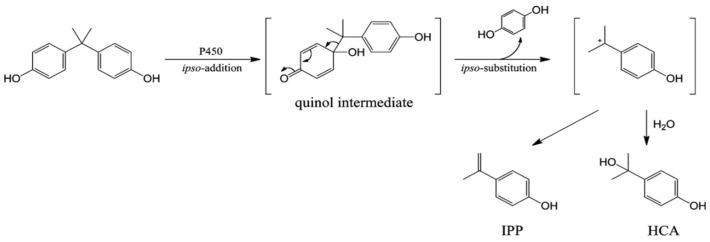
The metabolic pathway of BPA through ipso-substitution.

Despite extensive research on the effects and toxicity of BPA on the different kinds of receptors, little is known about the activity of most BPA analogs, especially those that have recently been applied as new, less toxic BPA substitutes. Among the various bisphenol analogs, bisphenol Z (BPZ) has emerged as a compound of interest due to its high structural rigidity and potent bioactivity. BPZ, like BPA, undergoes metabolic activation via hepatic cytochrome P450 enzymes. Notably, studies have demonstrated that its oxidative metabolites, such as those formed via ipso-substitution pathways (Figure 2), may exhibit even greater binding affinities towards estrogen receptors than their natural compounds [4,5]. This interaction contributes to endocrine disruption, particularly through interference with estrogen biosynthesis and metabolism.

**Figure 3:**
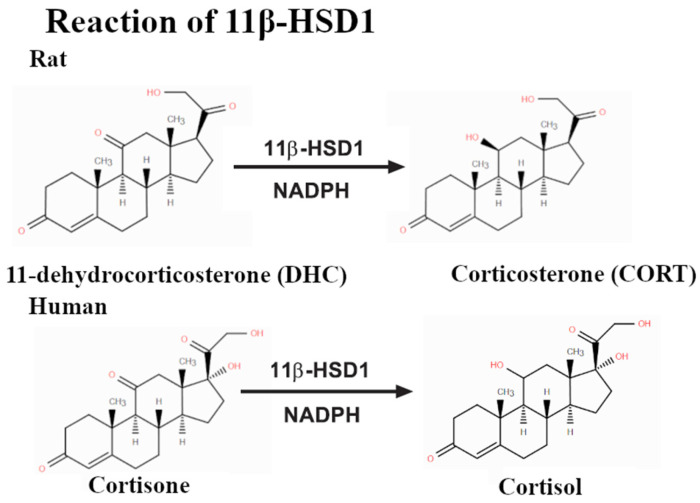
Reactions catalyzed by 11β-HSD1 result in the hydrogenation of the ketone group near C7.

In addition to their estrogenic effects, bisphenols affect other nuclear receptors. Of particular concern is their influence on glucocorticoid receptors (GRs) and related metabolic pathways. The enzyme 11β-hydroxysteroid dehydrogenase type 1 (11β-HSD1), which converts inactive cortisone to active cortisol (Figure 3), is sensitive to bisphenol interference [6,7]. Exposure to bisphenols, such as BPA and its analogs, has been shown to alter GR-related gene expression and increase local cortisol bioavailability, which may contribute to metabolic disorders such as obesity, hypertension, and insulin resistance [3,8,9].

Understanding the precise mechanisms of bisphenol-mediated enzyme modulation requires rigorous kinetic studies. The Michaelis-Menten model provides a foundational framework for interpreting enzyme behavior under varying substrate concentrations. However, the model has notable limitations, particularly in dynamic biological systems where non-Markovian behaviors and enzyme conformational changes may dominate [10].

To refine the kinetic characterization, graphical methods, such as the Lineweaver-Burk, Eadie-Hofstee, and Hanes-Woolf plots, are frequently employed [11,12,13,14,15]. These approaches facilitate the identification of different types of enzymatic inhibition, including competitive, non-competitive, and uncompetitive inhibition, which is crucial for explaining the modulatory effects of bisphenols on steroidogenic and detoxifying enzymes. Furthermore, chromatographic techniques, notably high-performance liquid chromatography (HPLC), are essential for the separation and quantification of reaction products, such as corticosterone, a downstream product of 11β-HSD1 activity. By coupling enzymatic assays with chromatographic analysis, the extent of bisphenol-induced inhibition can be evaluated in situ, thereby linking kinetic findings with biochemical outcomes.

**Figure 4:**
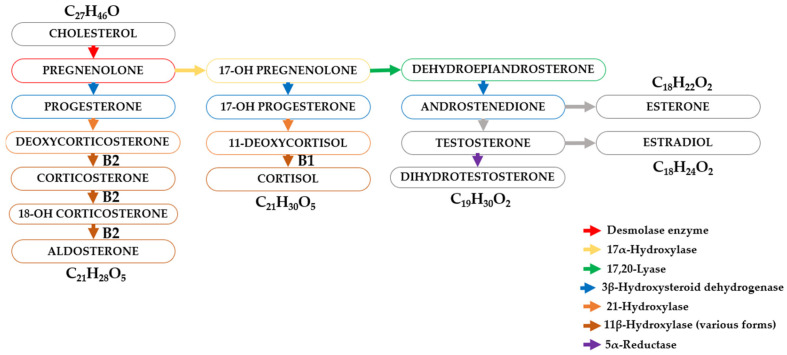
Schematic diagram of the transformation of steroid hormones (steroids).

Cholesterol is the precursor to several classes of steroid hormones, which include glucocorticoids (including cortisol), mineralocorticoids (aldosterone), androgens (e.g., testosterone), and estrogens (Figure 4).

These hormones exert their effects by binding to intracellular receptors, and these receptor–ligand complexes exert a profound influence on a variety of physiological processes. The adrenal cortex, a major source of steroid hormones, is divided into anatomically and functionally distinct zones. Cells in the zona glomerulosa, zona fasciculata, and zona reticularis synthesize mineralocorticoids, glucocorticoids, and androgens, respectively. The synthesis of glucocorticoids (cortisone and cortisol (hydrocortisone)) requires the conversion of progesterone by 17α-hydroxylase to 17α-hydroxyprogesterone, which is then converted to cortisol and cortisone by 21β-hydroxylase and 11β-hydroxylase (Figure 4).

In naturally occurring hormones, the effects of mineralocorticoids and glucocorticoids are not completely separated; some glucocorticoids significantly affect water and electrolyte balance. In fact, hydrocortisone and aldosterone stimulate mineralocorticoid receptors to the same degree. However, in mineralocorticoid-sensitive tissues, such as the kidneys, the action of 11β-hydroxysteroid dehydrogenase type 2 (11β-HSD2) converts hydrocortisone to an inactive metabolite of cortisone, thus protecting the receptor from inappropriate activation. Interestingly, in 1949, cortisone was identified in studies by Hench et al. as a hormone with potent anti-inflammatory effects [16,17,18,19]. Hench used cortisone first in patients with rheumatoid arthritis and then in rheumatic fever and showed remarkable efficacy in what had hitherto been inexorable inflammatory disorders [16,17,18,19].

This apparent anomaly results from the fact that the 11β-hydroxysteroid dehydrogenase isoform present in some tissues converts this hormone back into cortisol (i.e., hydrocortisone), thereby restoring its biological activity.

Irregularities in the biosynthesis of steroid hormones and their receptors can cause a variety of physiological abnormalities, which can, in turn, lead to clinical abnormalities. Hydrocortisone (cortisol), the main glucocorticoid in humans, is produced in the adrenal cortex and plays a crucial role in many physiological processes. Hydrocortisone is metabolized mainly by the 11β-hydroxysteroid dehydrogenase (11β-HSD), of which two isoforms (11β-HSD1 and 11β-HSD2) have been described. 11β-HSD2, which inactivates cortisol to cortisone, is localized mainly in the kidneys and the placenta, while 11β-HSD1 converts cortisone to cortisol in the liver and fat tissue. Both cortisol and cortisone are then catabolized by 5α- and 5β-reductases in the liver and converted into tetrahydro- and allo-tetrahydro-metabolites [20,21,22].

Disturbances in cortisol secretion can lead to life-threatening conditions such as Addison’s disease (primary adrenal insufficiency resulting in cortisol deficiency), which is characterized by muscle weakness, low blood pressure, depression, anorexia, weight loss, and hypoglycemia.

Cushing’s syndrome may be caused by excessive release of glucocorticoids from the adrenal glands (excess in cortisol release) or long-term use of glucocorticoids. The most common side effects of Cushing’s syndrome include euphoria (sometimes depression or psychotic symptoms and emotional lability), a moon-shaped face with red (congested) cheeks, buffalo hump, thinning of the skin, thin limbs, muscle loss, osteoporosis, tendency to hyperglycemia, negative nitrogen balance, increased appetite, accumulation of abdominal fat, obesity, increased susceptibility to infection, impaired wound healing, easy bruising, and other less common side effects (benign intracranial hypertension, cataracts, or avascular necrosis of the femur).

Also, alterations in levels of glucocorticoids in biological material, such as urine and/or in plasma, were observed in many other clinical conditions, e.g., in apparent mineralocorticoid excess (AME) syndrome [16,20,23], polycystic ovary syndrome (PCOS) [16,23], pre-eclampsia (PE) [16,23], hypertension [16,23], obesity and insulin resistance [16,23], and mental disorders, such as depression, bipolar disorder, and schizophrenia [16,23]. These other clinical conditions are not directly caused by the dysfunction of the adrenal glands.

Glucocorticoids are released into the blood in a pulsatile manner and are always present in the blood. In healthy individuals, their concentration in the blood is highest in the morning and gradually decreases throughout the day, reaching their lowest levels in the evening and at night. ACTH secretion is regulated by corticotropin-releasing factor (CRF), released from the hypothalamus, and vasopressin, released from the posterior pituitary. The release of both ACTH and CRF is, in turn, inhibited by negative feedback from increasing glucocorticoid concentrations in the blood. This functional hypothalamic–pituitary–adrenal system is referred to as the “HPA axis.” Thus, patients with an apparent mineralocorticoid excess (AME) syndrome have intense mineralocorticoid receptor activation by cortisol within specific cells of the kidney (within the cells of the distal nephron) but without a change in systemic cortisol levels, an intracrine effect.

Therefore, for the correct diagnosis of diseases, it is extremely important to test for the correct concentrations of the following compounds in biological materials: plasma or serum cortisol-to-cortisone ratio, urinary free cortisol to cortisone, or the sum of A-ring reduced metabolites of cortisol (tetrahydrocortisol and allo-tetrahydrocortisol) to an A-ring reduced metabolite of cortisone (tetrahydrocortisone), expressed as the ratio (tetrahydrocortisol + allo-tetrahydrocortisol)/tetrahydrocortisone. The concentrations and ratios of the determined analytes in plasma and urine are influenced by the impact of the function of 11β-HSD1 and 11β-HSD2, which are responsible for the interconversion of cortisol and cortisone. Moreover, the (tetrahydrocortisol + allo-tetrahydrocortisol)/tetrahydrocortisone ratio is influenced by the activities of 5α- and 5β-reductases, which catalyze the production of A-ring reduced metabolites of both cortisol and cortisone [16,20,23].

In tissues such as the liver, adipose, and adult brain tissue, 11β-HSD2 is absent, but there is abundant 11β-HSD1. 11β-HSD1 catalyzes the reaction in intact cells and organs, thus regenerating active cortisol from inert cortisone and amplifying the local glucocorticoid signal, particularly at glucocorticoid receptors. Glucocorticoid receptors have 10-fold lower affinity for cortisol than mineralocorticoid receptors and are thus partially unoccupied by cortisol at physiological concentrations, allowing a dynamic range for 11β-HSD1 amplification inside cells to impact signaling. In contrast, mineralocorticoid receptors are largely occupied by physiological cortisol concentrations where 11β-HSD2 is absent, so 11β-HSD1 may have less impact on signaling via mineralocorticoid receptors [16].

Inhibition of 11β-HSD1 is a plausible target for treating metabolic diseases, atherosclerosis, and disorders of cognition with aging. The effects of selective 11β-HSD1 inhibitors in humans with diabetes and metabolic disease have been modest to date, at least for the chosen therapeutic target of lowering markers of glycemic control or blood pressure, and the utility of such inhibitors in clinical therapy remains to be established. Perhaps the main indication might be in early metabolic disease to ameliorate multiple risk factors. The effect on atherosclerosis is crucial, the main final cause of mortality in the obesity–diabetes–metabolic syndrome continuum.

**Figure 5:**
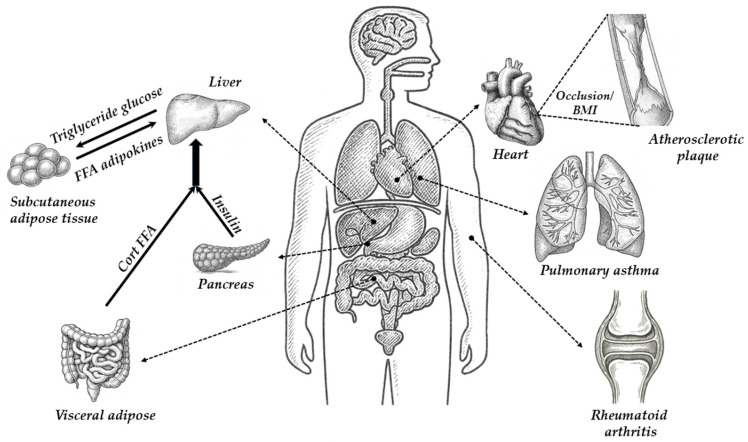
The most important human organs in which the functioning of 11β-hydroxysteroid dehydrogenase type 1 (11β-HSD1) may be most significant.

Figure 5 shows the most important human organs in which the functioning of 11β-hydroxysteroid dehydrogenase type 1 (11β-HSD1) may be most important. The drawing refers to possible effects of 11β-HSD1 inhibition or deficiency on various organs and diseases, including obesity, diabetes, metabolic diseases, cardiovascular disease and atherosclerosis, and age-related brain changes, and the potential impact on asthma and inflammation.

The potential role of 11β-HSD1 and 11β-HSD2 in metabolic organ interrelationships and related diseases was described in detail by Chapman, Holmes, and Seckl [16]. The potential role of 11β-HSD1 in metabolic organ interrelationships in obesity is described briefly below. In patients with normal weight health, 11β-HSD1 acts largely as a reductase. 11β-HSD1 performs an important endocrine role in the splanchnic bed by contributing to 40% of daily glucocorticoid production. In addition, 11β-HSD1 has intracrine actions, amplifying the glucocorticoid signal inside hepatocytes, β and α cells of pancreatic islets, and adipocytes. In patients with modest obesity, 11β-HSD1 is elevated in adipocytes and β cells but not in hepatocytes. This increases insulin resistance in adipose tissues, increases the release of pro-inflammatory (antimetabolic) adipokines, and increases portal blood glucocorticoid and fatty acid delivery to the liver. It also increases islet insulin release to glucose, plausibly without changing hepatic insulin sensitivity [16]. In patients with severe obesity, greater rises in adipose and β-cell 11β-HSD1 lead to a failure of pancreatic insulin release, worsening peripheral insulin resistance and metabolic disease despite potentially “compensatory” declines in hepatic glucocorticoid regeneration, which might even contribute to HPA axis activation due to the loss of bulk glucocorticoid regeneration. 11β-HSD1 inhibition maximizes subcutaneous adipose and liver insulin sensitivity and visceral adipose AMPK signaling and reduces inflammation and portal glucocorticoid and fatty acid release. Despite reduced beta cell insulin release and HPA axis activation (without elevation of glucocorticoid levels), the balance of metabolism favors reduced hepatic gluconeogenesis, increased beta-oxidation of fats, “safe” calorie storage in subcutaneous adipose deposits, reduced visceral adipose mass, and metabolic health [16].

The impact of 11β-HDSD1 inhibition or deficiency on atherosclerosis by reducing multiple risk factors and atherosclerotic lesion size was described by Chapman, Holmes, and Seckl [16]. The probable impacts of 11β-HDSD1 inhibition or deficiency on atherosclerosis are possible by reduced atherosclerotic risk factors (lower blood glucose and insulin, lower LDL cholesterol, higher HDL cholesterol); reduced tissue lipid delivery and uptake; reduced lesion size; reduced lesion inflammation; possible reduction in thrombosis; and increased angiogenesis and collateral formation [16]. If a myocardial infarction occurs, the angiogenic effects of 11β-HSD1 deficiency or inhibition promote angiogenesis and recovery of myocardial function for the same initial infarct size.

Also, it has been demonstrated that glucocorticosteroids can be produced locally, outside the adrenal glands, including in the thymus and skin. This may be particularly important in local control of the inflammatory process, e.g., rheumatoid arthritis.

11β-HSD1 is widely and unevenly distributed in the adult CNS. Although 11β-HSD1 is highly expressed in the cerebellum, hippocampus, and cortex, there is a discrete microdistribution within these areas. People exposed to chronically high glucocorticoids (endogenous or exogenous) are susceptible to cognitive, affective, and, rarely, psychotic disorders. Individual differences in cognitive function with aging are associated with stressful events or rising cortisol levels across a lifespan. 11β-HSD1 inhibition as a potentially tractable approach to cognitive decline in aged humans was examined. Treatment of elderly healthy men (aged 65–69 years) with carbenoxolone for 4 weeks, plus amiloride to block the adverse effects of renal 11β-HSD2 inhibition, in a double-blind, placebo-controlled, randomized study, led to cognitive improvements in verbal memory, a hippocampus-associated function [24]. Similar effects were found in middle-aged subjects with type 2 diabetes. The findings of another paper implied possible causal roles of elevated 11β-HSD1 in adverse cognitive and brain structural effects with aging, especially cognitive decline over the age of 60 years [25].

The mechanisms by which 11β-HSD1 becomes elevated in specific cells in the aging brain, bones, ovaries, immune system, and perhaps adipose tissue are not fully understood. Whatever the mechanism and its biological importance, 11β-HSD1 and its elevation in specific tissues, in association with pathogenesis, offer an opportunity for therapy and, therefore, should be further examined.

11β-HSD2 inactivates cortisol to cortisone and is localized mainly in the kidneys and the placenta, while 11β-HSD1 regenerates cortisone to cortisol in the liver and fat tissue. Both cortisol and cortisone are then catabolized by 5α- and 5β-reductases in the liver and converted into tetrahydro- and allo-tetrahydro-metabolites. A diminished function of 11β-hydroxysteroid dehydrogenase 2 (11β-HSD2) was found in placentae from preeclamptic pregnancies. Kosicka et al. described a study in which they aimed to answer the question of whether the functions of primary enzymes involved in cortisol metabolism, namely, 11β-HSD1, 11β-HSD2, and 5-reductases (both 5α- and 5β), are altered in the course of hypertensive pregnancy [20,23]. The main findings described by them included markedly intensified cortisol metabolism manifested in the increased function of renal 11β-HSD2 and 5α- and 5β-reductases in pre-eclampsia, as well as an enhanced function of renal 11β-HSD2 and 5β-reductase in gestational hypertension [20,23]. The authors concluded that the glucocorticoid balance in pre-eclampsia is clearly shifted towards decreasing cortisol concentration either due to the intensified conversion of cortisol to cortisone or enhanced production of tetrahydro- and allo-tetrahydro-metabolites [20,23].

Kosicka et al. found significant differences in glucocorticoid balance in pregnancy-related hypertension [20]. Plasma cortisol to cortisone was significantly lower in patients with pre-eclampsia than in controls (3.00 vs. 4.79; *p* < 0.001). Increased function of renal 11β-HSD2 in gestational hypertension and gestational hypertension was manifested by a significantly lower urinary free cortisol-to-cortisone ratio (0.169 and 0.206 vs. 0.277 in controls; *p* < 0.005) [20]. Markedly enhanced metabolism of cortisol was observed in pregnancy-related hypertension, with no significant alterations in chronic hypertension, and the changes were more clearly expressed in gestational hypertension than in gestational hypertension [20].

Bisphenols’ effects on 11β-HSD1 remain unclear. The inhibitory effects of bisphenols are either unknown on 11β-HSD1 or have been partially explained for some of the bisphenol A analogs. Wang et al. described the inhibitory effects of TBBPA, MCBPA, TrCBPA, TCBPA, TBBPS, and BPS on 11β-HSD1 [26]. All these BPA derivatives are mixed/competitive inhibitors of both human and rat enzymes (11β-HSD1) [16]. In another study, Wang et al. also described the inhibitory effects of BPA, BPG, BPH, BPAME, DABPA, and TMBPA on 11β-HSD1 [27]. The examined derivatives of BPA are also mixed/competitive inhibitors of both human and rat enzymes (11β-HSD1) [27].

Usually, BPA alternatives are structurally related to BPA and also have endocrine-disrupting effects. BPA alternatives are, however, not necessarily less estrogenic than BPA in human breast cancer cells [28]. For example, BPAF, BPB, and BPZ are more estrogenic than BPA. BPA and 17-beta estradiol bind in a similar manner, with two phenol rings pointing to both ends of the estrogen receptor hydrophobic pocket. Differences in estrogenic activities between the different analogs of BPA applied as alternatives may be due to the different groups present at the bridge between the two phenol rings. Their hydrophobicity drives their affinity since the matching of the group carried by the methylene bridge is known to determine binding affinity toward the hydrophobic surface of the estrogen receptor binding site. Such a mechanism of estrogen receptor interaction is supported by the hydrophobicity ranking of bisphenols (log P values BPZ > BPAF > BPAP > BPB > BPF > BPS), which corresponds to their AC50 toxicity score [28]. Estrogenicity was assessed by cell growth in an estrogen receptor-mediated cell proliferation assay and by the induction of the estrogen response element (ERE)-mediated transcription in a luciferase assay. BPAF was the most potent bisphenol (AC50 = 0.08 μM) at simulating ERE-luciferase reporter gene expression, followed by BPB (AC50 = 0.3 μM) > BPZ (AC50 = 0.4 μM); similarly, BPA (AC50 = 0.4 μM) > BPF and BPAP (AC50 = 1 μM) > BPS (AC50 = 1.5 μM) have estrogenic effects in the same range as BPA. However, the estrogenic effects of BPB and BPZ were not completely antagonized, suggesting an estrogenic activation mechanism independent of the estrogen receptor [28].

The newest BPA analogs used as alternatives are still the subject of research, including the latest ones [29,30]. BPZ exposure compromises early embryo quality by disrupting mitochondrial function, triggering oxidative stress and DNA damage, influencing histone modification and maternal-to-zygotic transition, and ultimately inhibiting early embryonic development [29]. In vitro analysis of primary Leding cells demonstrated that BPZ heightened oxidative stress and diminished testosterone production [30]. In a published paper, Zhang et al. concluded that compared with BPE, BPF, and BPA, BPFL has a stronger inhibition against human 11β-HSD2, which may be due to the fact that BPFL contains a large-sized fluorene group, while BPE, BPF, and BPA only contain hydrogen or methyl groups [31]. The increase in the size of the substituted alkane group in the methane moiety of the bridge of BPs seems to increase its inhibitory potency. In this case, the inhibitory potency follows the size of the substituted alkanes: BPFL (fluorenyl) > BPAP (phenyl and methyl) > BPZ (hexanyl) > BPB (methyl and ethyl) > BPA (two methyl) > BPE (methyl and hydrogen) =  BPF (two hydrogens) [31]. Apparently, the increase in the size of hydrocarbon substitution in BPAP (phenyl group) and BPZ (cyclohexyl group) also leads to increased inhibitory potency, with IC50 values of 4.92 ± 0.17 and 9.40 ± 0.43 μM, respectively [31].

The results should be further confirmed by more experiments conducted by various research centers. Bisphenol Z is one of the newest bisphenol A analogs, the toxicity and other properties of which are not sufficiently explained. This study aimed to characterize the inhibitory effects of BPZ on 11β-HSD1 activity in the transformation of 11-dehydrocorticosterone (DHC) to corticosterone (CORT) by integrating enzymatic kinetics, inhibition modeling, and chromatographic quantification of the reaction product, corticosterone (CORT). This study contributes to the broader understanding of bisphenol Z, an underexplored bisphenol analog, within the context of its effect on glucocorticoid regulation, a target less commonly assessed in bisphenol safety and inhibitory effects, such as the aforementioned bisphenol Z.

## 2. Results

### 2.1. Determination of Corticosterone by HPLC-DAD

**Figure 6:**
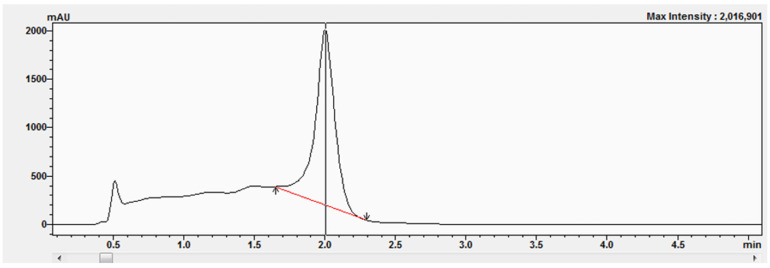
Chromatogram of the corticosterone standard at a concentration equal to 577 μM.

**Figure 7:**
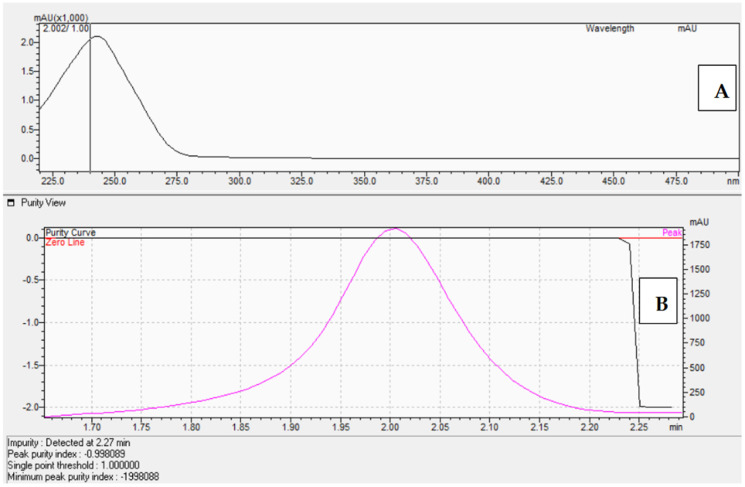
UV-Vis spectrum in the range of 220 nm–280 nm (**A**) and peak purity index ((**B**), equals 0.998) of the corticosterone standard at 577 μM.

The corticosterone (CORT) standard was chromatographed on an Acquity UPLC BEH C18 column (50 × 2.1 mm, 1.7 μm) from Waters using an eluent system composed of 0.1% formic acid in water (solvent A) and 0.1% formic acid in acetonitrile (solvent B), applied in a gradient system as described in Section 4.3. The presence of the corticosterone standards was confirmed by comparing retention times (Figure 6) as well as by UV-Vis analysis (Figure 7A) and determining the peak purity index (Figure 7B).

The peak purity index is a numerical measure of the quality of coincidence between two datasets. A peak purity index of 1 indicates that the compared spectra are identical and the marked substance peak does not contain impurities. The optimized chromatographic system with a mobile phase gradient of up to 8 min allowed for the determination of the corticosterone peak at approximately 2 min (Figure 6).

**Figure 8:**
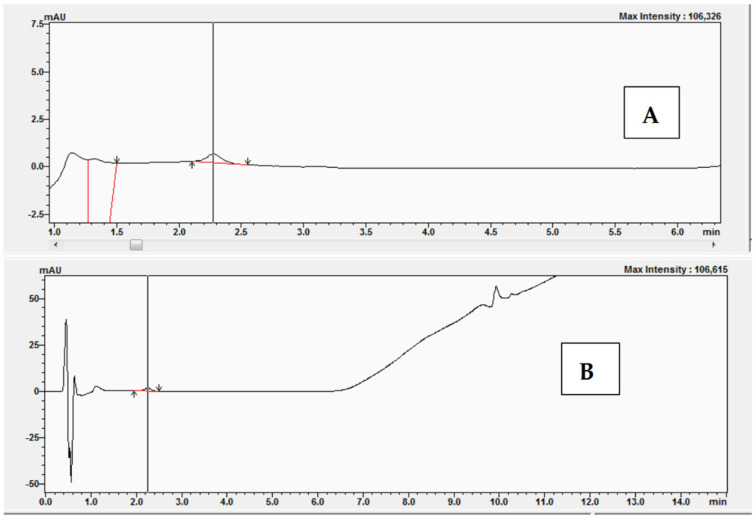

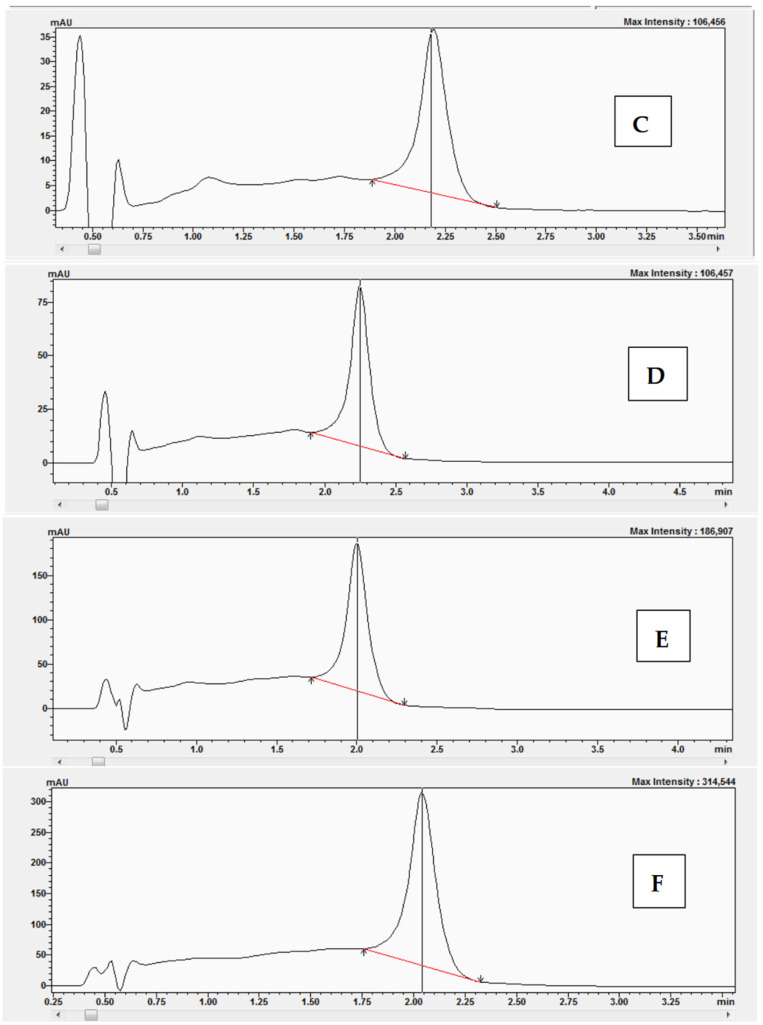
Chromatograms obtained after HPLC-DAD analysis with corticosterone at the following concentrations: (**A**) 0.1 μM, (**B**) 0.5 μM, (**C**) 5 μM, (**D**) 25 μM, (**E**) 50 μM, and (**F**) 100 μM.

An optimized chromatographic system using gradient elution was used to determine corticosterone during chromatographic analyses, the results of which were used to determine the Lineweaver-Burk relationship and others. A calibration curve was performed based on the peak areas of standard solutions of corticosterone (Figure S1). Examples of the chromatograms (Figure 8A–F) obtained during HPLC-DAD at the following concentrations are presented for 0.1 μM (Figure 8A), 0.5 μM (Figure 8B), 5 μM (Figure 8C), 25 μM (Figure 8D), 50 μM (Figure 8E), and 100 μM (Figure 8F).

### 2.2. Kinetic Study—Inhibitory Effects of Bisphenol Z on 11β-Hydroxysteroid Dehydrogenase 1

**Figure 9:**
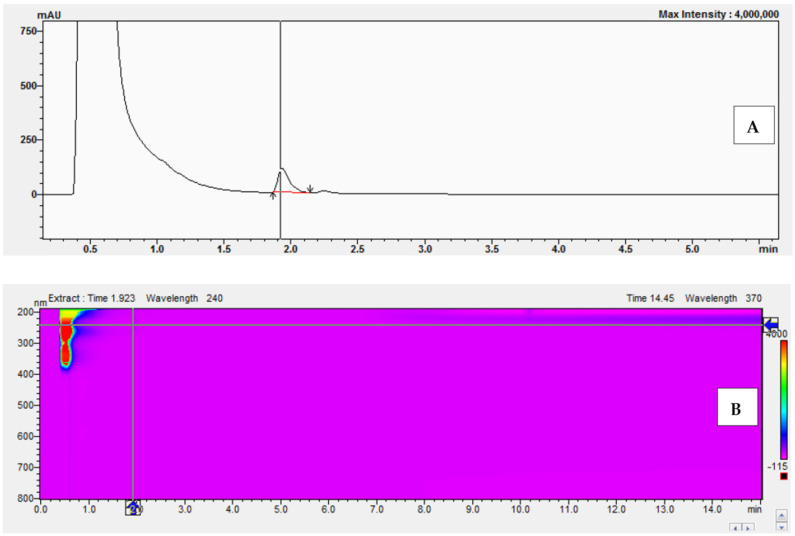
Chromatogram (**A**) and UV-Vis analysis (**B**) obtained after the conversion of 11-dehydrocorticosterone (DHC) to corticosterone (CORT) during kinetic studies without BPZ (0 μM) and with DHC at 2.5 μM.

A characteristic of enzymes that obey Michaelis-Menten kinetics is that suitable inhibitors can compete with substrates for the enzyme active site, thus impeding the reaction. If the inhibitor binds reversibly to the enzyme active site, then the substrate can compete for the active site, leading to complete inhibition. The experiments began with the preparation of a reaction mixture without bisphenol Z. The experiments were performed with the following concentrations of 11-dehydrocorticosterone (DHC): 1 μM, 2 μM, 2.5 μM, and 3 μM. Figure 9 shows examples of the chromatograms and UV-Vis analysis obtained after the conversion of 11-dehydrocorticosterone (DHC) to corticosterone (CORT) during the kinetic studies without BPZ (0 μM) and with DHC at a concentration of 2.5 μM.

**Figure 10:**
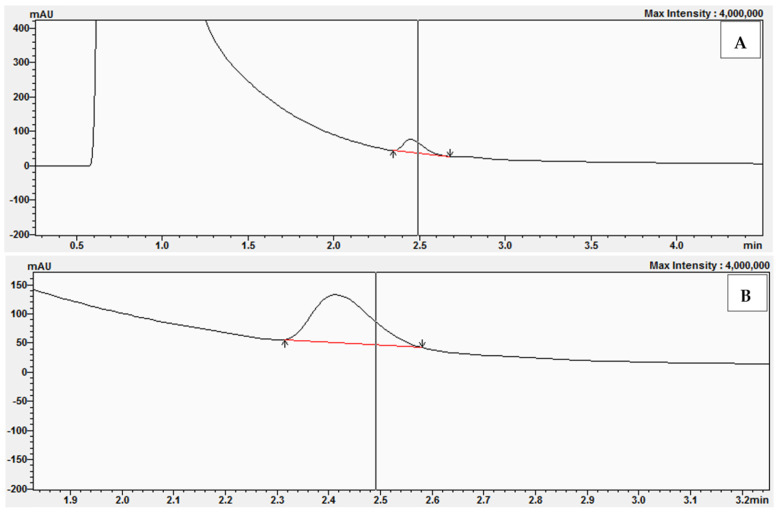

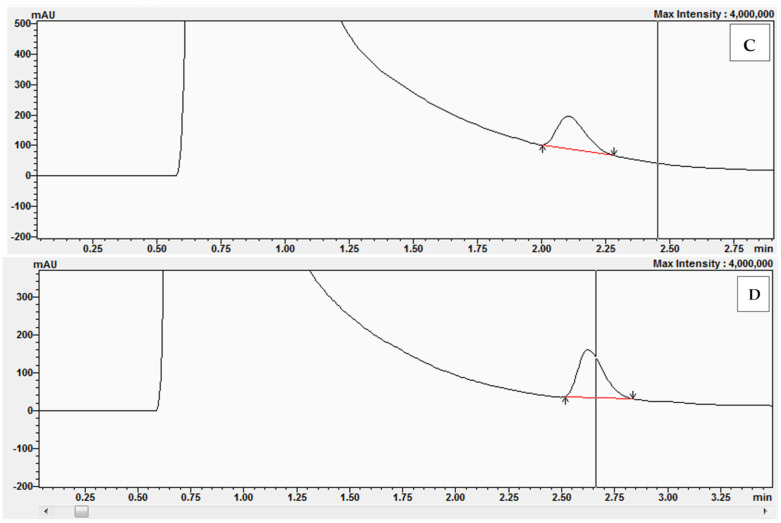
Chromatograms obtained after the conversion of 11-dehydrocorticosterone (DHC) to corticosterone (CORT) during kinetic studies with BPZ at 10 μM and with DHC at the following concentrations: 1 μM (**A**), 2 μM (**B**), 2.5 μM (**C**), and 3 μM (**D**).

In the next series of experiments, we prepared reaction mixtures containing the following concentrations of BPZ: 0, 5, 10, 20, and 30 μM. Examples of the chromatograms obtained after conversion of 11-dehydrocorticosterone (DHC) to corticosterone (CORT) during the kinetic studies with a concentration of BPZ at 10 μM and with DHC at the concentrations of 1 μM (A), 2 μM (B), 2.5 μM (C), and 3 μM (D) are presented in Figure 10. During the experiments with reaction mixtures containing 20 μM and 30 μM, complete inhibition by BPZ was observed on 11β-hydroxysteroid dehydrogenase 1 (11β-HSD1).

## 3. Discussion

The type of inhibition can be determined by graphically analyzing the Lineweaver-Burk relationship. As a result, we can distinguish three types of inhibition in the Lineweaver-Burk plots for different inhibitor concentrations:

- Intersection on the Y-axis and forming a bunch, which indicates competitive inhibition, meaning the inhibitor competes with the appropriate substrate for the enzyme’s active site;
- Intersection on the X-axis, meaning the inhibitor binds to the enzyme at a site other than the active site, altering the properties of the enzyme molecule so that it cannot catalyze a specific enzymatic reaction;
- No intersection on any axis, neither the y-axis nor the x-axis, only between them, meaning mixed inhibition, in which the inhibitor can bind both to the active site of the enzyme and to another site on the enzyme molecule, in both cases “blocking” its activity.

### 3.1. Kinetic Analysis of 11β-Hydroxysteroid Dehydrogenase 1 (11β-HSD1) Inhibition by Bisphenol Z (BPZ)

**Figure 11:**
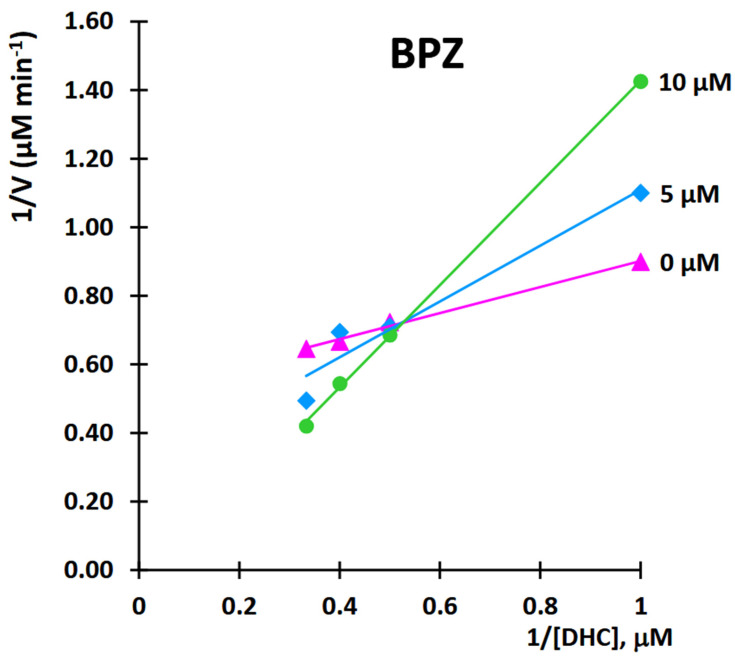
Kinetic analysis of 11β-hydroxysteroid dehydrogenase 1 (11β-HSD1) inhibition by bisphenol Z (BPZ). Lineweaver-Burk plot of 11β-hydroxysteroid dehydrogenase 1 (11β-HSD1) activity over a range of the substrate—11-dehydrocorticosterone (DHC) concentration for bisphenol Z (BPZ).

**Figure 12:**
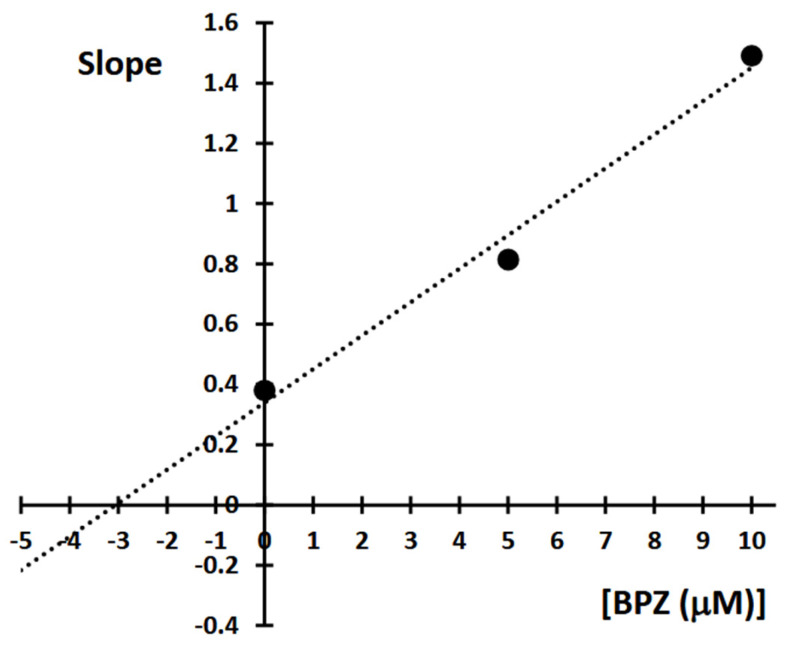
Determination of 11β-hydroxysteroid dehydrogenase 1 (11β-HSD1) inhibition constants (Ki) by plotting the slope of the primary Lineweaver-Burk plot vs. bisphenol Z (BPZ) concentration.

This study aimed to characterize the inhibitory effects of BPZ on 11β-HSD1 activity in the transformation of 11-dehydrocorticosterone (DHC) to corticosterone (CORT). The mode of inhibition and value of the inhibition constant (Ki) of BPZ were investigated using Lineweaver-Burk plots (Figure 11 and Figure 12).

The obtained results showed that BPZ exhibits mixed inhibition behavior. The Ki value was estimated from the replots of the slope of the individual Lineweaver-Burk plots versus the inhibitor concentrations (Figure 10). The intersection of the plot lines occurred outside both the x- and y-axes, indicating a mixed-type inhibition of 11β-HSD1 activity (Figure 9). This suggests that BPZ can bind to both the active site and an allosteric site on the enzyme, thereby only partially influencing the binding of the substrate in the active site. The Ki value for 11β-HSD1 inhibition by BPZ was estimated at 3 μM, based on the secondary replot derived from the Lineweaver-Burk plots. Our results are consistent with those published by Lu et al. [7], whose results indicate a competitive or mixed type of inhibition of bisphenols (including BPZ) on the rat and human 11-hydroxysteroid dehydrogenase 1 enzyme.

**Figure 13:**
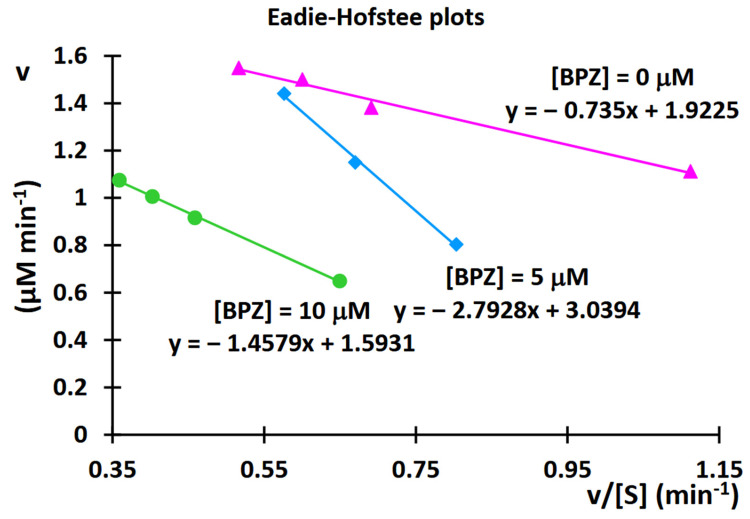
Eadie-Hofstee plot after kinetic analysis of 11β-hydroxysteroid dehydrogenase 1 (11β-HSD1) inhibition by bisphenol Z (BPZ).

By varying the concentration of the substrate (11-dehydrocorticosterone (DHC)) for each amount of inhibitor (BPZ), the resulting saturation curves were compared using an Eadie-Hofstee plot (Figure 13).

As with the Lineweaver-Burk relationship, analyzing the Eadie-Hofstee plots can also determine the type of inhibition. If the Eadie-Hofstee plots for different inhibitor concentrations intersect only between the y- and x-axes, but do not intersect either axis, y or x, the inhibition is mixed. In mixed inhibition, the inhibitor can bind both to the enzyme’s active site and elsewhere on the enzyme molecule, in both cases acting as an inhibitor, reducing its activity.

When the saturation curves intersect on the y-axis, it signifies that at infinite substrate concentrations, the maximum reaction velocity is independent of the amount of the inhibitor, which confirms that competitive inhibition is indeed operating [14,15]. In our case, where the saturation curves intersect on the y-axis or between the y- and x-axes, this may confirm that a mixed mode of inhibition is indeed operating. The experiments were performed with the greatest possible care and were repeated four to eight times for each DHC or BPZ concentration. During the initial experiments, the reaction mixture volume was 100 microliters. To obtain reproducible results, the reaction mixture volume was increased to 500 microliters (the results are described in this paper). The discrepancies observed may be due to the increased pressure during chromatographic analysis and experiments with the reaction mixture containing rat microsomes. Therefore, chromatographic systems with a reduced velocity of the mobile phase flow were applied. Initially, a mobile phase gradient was applied at 0.4 mL per minute; subsequently, in some experiments, the mobile phase flow was reduced to 0.35 or 0.25 mL/min.

Lu et al. [7] and Zhang et al. [31] obtained very interesting results, including the Lineweaver-Burk relationships. Compared to the results published by Lu et al. [7], we obtained a mixed type of inhibition in our study. We presented raw chromatogram data showing the reaction product during the determination of the Lineweaver-Burk and Eadie-Hofstee relationship. We could not find a publication in which the authors presented Eadie-Hofstee plot relationships. Also, we could not find any other publications where the results were presented as raw data with chromatograms, spectra, and other raw data during the determination of the Lineweaver-Burk and Eadie-Hofstee relationship. Our publication presents results that were optimized for a 500 μL reaction mixture, resulting in reproducible results (compared to results not published with a 100 μL reaction mixture). In the described experiments, the authors applied significantly smaller volumes of the reaction mixture [7]. In their publications, the results were performed using UPLC-MS/MS [7,31]. HPLC-DAD analyses are much less expensive than UPLC-MS/MS. We also used a different chromatographic system and a different mobile phase gradient to determine the reaction product during kinetic studies. The authors presented very entertaining and valuable results, but without chromatograms [7,31].

### 3.2. Comparison of Lineweaver-Burk, Eadie-Hofstee, and Hanes-Woolf Plots

Table 1 presents a comparison of the Lineweaver-Burk, Eadie-Hofstee, and Hanes-Woolf relationship graphs, and the commentary describes the strengths and weaknesses of each of the equations describing them. Results presented in the form of various relationships always have advantages, but, unfortunately, also limitations. Like other straight-line forms of the Michaelis-Menten equation, the Eadie-Hofstee plot was used historically for rapid evaluation of the parameters *K_m_* and *V*, but it has been largely superseded by nonlinear regression methods that are significantly more accurate and no longer computationally inaccessible. As the ordinate scale spans the entire range of theoretically possible *v* values, upon inspecting an Eadie-Hofstee plot, one can see how well the experimental data fill the theoretical area of a relationship.

Results presented in the form of Eadie-Hofstee plots also have certain limitations. These restrictions apply to the following experiments:

- Most [S] values are too small, with a substrate that is not soluble enough or too expensive to use concentrations above *K_m_*. In this case, *V*/*K_m_* cannot be estimated satisfactorily.
- Most [S] values are too large, and a value concentrated above *K_m_*. In this case, *V* cannot be estimated satisfactorily.

### 3.3. Molecular Docking Results

Molecular docking showed that bisphenol Z forms energetically favorable complexes with all the examined enzymes (Table 2). For human 11β-HSD1, the estimated binding free energy was −8.21 kcal/mol, with a predicted inhibition constant of 953.93 nM. The rat 11β-HSD1 isoform exhibited slightly stronger binding (−8.29 kcal/mol, Ki = 839.63 nM), whereas bisphenol Z showed somewhat weaker interactions with Arabidopsis 11β-HSD2 (−8.06 kcal/mol, Ki = 1230 nM).

Molecular docking analyses demonstrated that bisphenol Z forms energetically favorable complexes with all the examined enzymes, as summarized in Table 2. For the human 11β-HSD1 isoform, the estimated binding free energy was −8.21 kcal/mol, with a predicted inhibition constant (Ki) of 953.93 nM. The rat 11β-HSD1 isoform exhibited slightly stronger binding (−8.29 kcal/mol, Ki = 839.63 nM), whereas bisphenol Z displayed somewhat weaker interactions with Arabidopsis 11β-HSD2 (−8.06 kcal/mol, Ki = 1230 nM). These data indicate that bisphenol Z can effectively interact with 11β HSD enzymes across species, with the highest predicted affinity observed for the rat isoform, closely followed by the human enzyme. The relatively weaker binding to the plant 11β-HSD2 suggests isoform- and species-specific variations in the binding pocket composition and architecture, which likely modulate the strength and nature of ligand–protein interactions.

**Figure 14:**
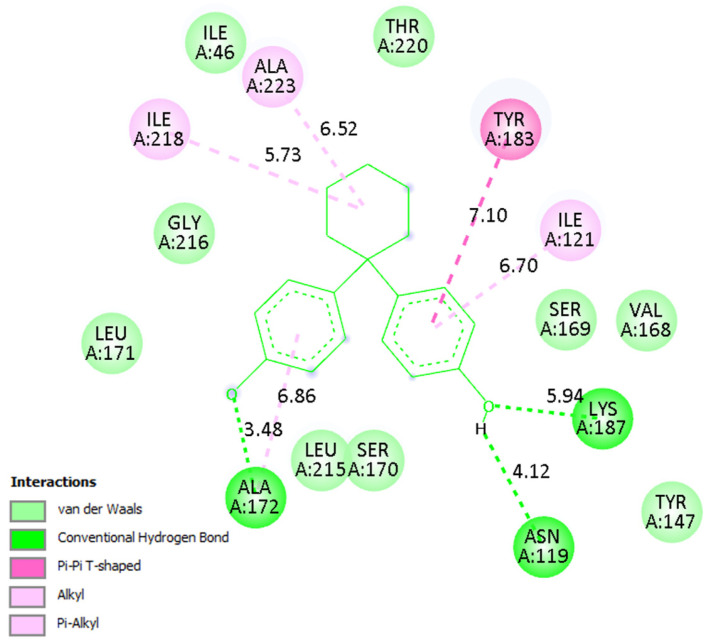
Binding mode of bisphenol Z in human 11β-HSD1. Left: 3D visualization showing the ligand as green sticks and the protein active site as a gray ribbon. Right: 2D interaction diagram indicating hydrogen bonds (green), π–π and π–alkyl interactions (purple), and van der Waals contacts (light green).

**Figure 15:**
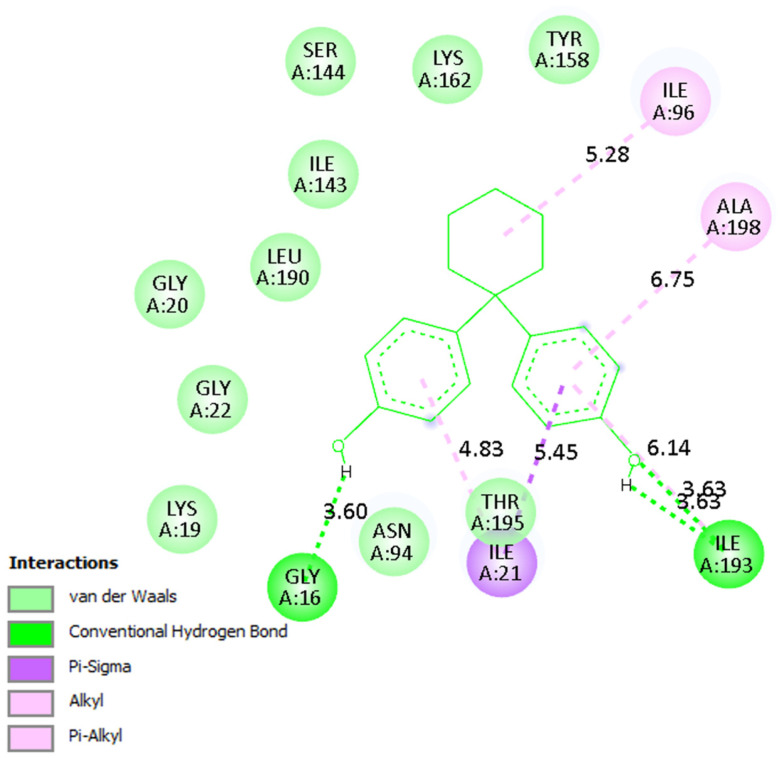
Binding mode of bisphenol Z in rat 11β-HSD1. Interaction networks show dominant hydrophobic and π-stacking contacts with key active site residues.

**Figure 16:**
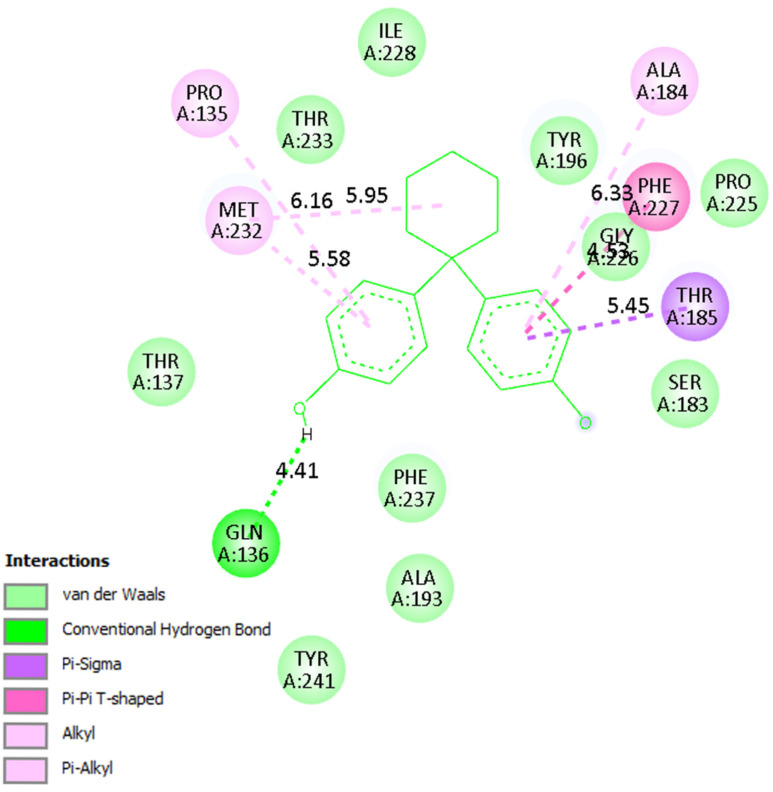
Binding mode of bisphenol Z in Arabidopsis 11β-HSD2. The ligand forms π-π T-shaped and π-sigma interactions with Phe227 and Tyr196, along with hydrogen bonds to Gln136 and Ser183.

A detailed analysis of the interaction profiles (Table 3, Figure 14, Figure 15 and Figure 16) revealed that bisphenol Z consistently occupies the active site cavities of all three enzymes, forming a stabilizing network of non-covalent interactions. Across all complexes, the binding mode is predominantly governed by van der Waals contacts, π-π stacking, and π–alkyl interactions, supplemented by a limited number of conventional hydrogen bonds. In the human 11β HSD1 complex, the aromatic rings of bisphenol Z engage in π-π stacking interactions with Tyr183, while hydrophobic contacts with Ile218, Leu215, and Ala223 provide additional stabilization. Moreover, hydrogen bonds with Asn119 and Lys187 enhance ligand anchoring, resulting in a rigid and energetically favorable conformation within the pocket. The rat 11β-HSD1 complex exhibits a similar interaction pattern, with π-π contacts involving Tyr158 and Ala198 and hydrogen bonds to Gly16 and Ile193, reflecting a high degree of structural complementarity between the ligand and the enzyme. In contrast, the Arabidopsis 11β-HSD2 complex presents a distinct interaction profile, likely reflecting species-specific active site features: bisphenol Z forms π-π T-shaped interactions with Phe227 and Tyr196, π-sigma contacts with Thr185, and hydrogen bonds with Gln136 and Ser183, collectively stabilizing its orientation within the binding cavity.

Overall, these findings underscore the critical role of hydrophobic and aromatic interactions—particularly π-π stacking with Tyr, Phe, and Ala residues—in driving bisphenol Z binding to 11β-HSD isoforms. The supplementary contribution of hydrogen bonds with polar residues (e.g., Asn, Gln, Lys) further refines ligand positioning within the pockets. Importantly, the minimal contribution of electrostatic interactions suggests that the binding mechanism of bisphenol Z is primarily dominated by hydrophobic and aromatic contacts, with subtle structural differences among isoforms influencing overall affinity.

In the present study, molecular docking was employed primarily as a complementary tool to rationalize the experimental findings obtained from enzyme kinetics. The docking analysis helped visualize how BPZ may interact with the active site of 11β-HSD1 and highlighted the possible contribution of hydrophobic contacts and π-π stacking interactions (e.g., Tyr183, Asn119 in human 11β-HSD1). However, we acknowledge that docking alone provides only a static representation of the binding event. Molecular dynamics (MD) simulations are widely recognized as a powerful approach to validate docking results and to explore the conformational flexibility and stability of protein–ligand complexes over time. Full-scale MD simulations were not performed in the present work, since our primary aim was to establish, through experimental enzyme kinetics, the inhibitory mechanism of BPZ. We consider MD simulations as an important next step that will further confirm the stability of BPZ-11β-HSD complexes and provide deeper insights into their dynamic behavior.

## 4. Materials and Methods

### 4.1. Chemicals and Instruments

NADPH (β-nicotinamide adenine dinucleotide phosphate, reduced form) and bisphenol Z (CAS No. 843-550, purity ≥ 99%) were purchased from Sigma-Aldrich (St. Louis, MO, USA). Corticosterone (CAS No. 50-22-6) was obtained from Steraloids, Inc. (Newport, RI, USA). HPLC-grade methanol, acetonitrile, dimethyl sulfoxide (DMSO) (catalog# 34869-500 mL), and water (LC-MS-grade) were obtained from Merck (Darmstadt, Germany). Glucose 6-phosphate sodium salt (CAS# 54010-71-8) was obtained from Santa Cruz Biotechnology, Inc. (Heidelberg, Germany). Liver microsomes from Sprague Dawley rats with a total protein concentration of 20 mg/mL were obtained from Thermo Fisher Scientific, Life Technologies (catalog# RTMCPL, 0.5 mL). Phosphate-buffered saline (PBS, pH 7.4) was prepared using commercially available ready-made PBS tablets (BIONOVO, Legnica, Poland, catalog# 092810305). One tablet was dissolved in 100 mL ultrapure water (LC-MS-grade) to produce a buffer containing 140 mM NaCl, 3 mM KCl, and 10 mM phosphate.

Solutions of bisphenol Z, corticosterone, glucose 6-phosphate sodium salt, NADPH, and phosphate-buffered saline, which is used to prepare the reaction mixtures, were stored at 5–8 °C in a refrigerator. Liver microsomes solutions were prepared on an ongoing basis, after completing the experiments for a specific BPZ concentration. The liver microsomes from the Sprague Dawley rats were stored in a deep freeze at −80 °C.

### 4.2. Enzyme Activity Assay

The activity of 11β-HSD1 was evaluated by tracking the reduction of dehydrocorticosterone (DHC) to corticosterone (CORT) using rat-derived 11β-HSD1. A reaction mixture volume of 500 μL was prepared, which included DHC (0, 1, 2, 2.5, and 3 μM), NADPH (2 μM), glucose-6-phosphate (G6P, 2 μM), the enzymatic source (1 μM rat liver microsomes), and BPZ (0 μM, 5 μM, and 10 μM). The mixtures were then incubated at 37 °C in a water bath for 15 min. The experimental procedures consisted of the following studies, which were run for 15 min each: a time-dependence study was performed with 0.33, 0.4, 0.5, and 1 μM for 1/[DHC], enzyme kinetics were determined for concentrations of 0, 1, 2, 2.5, and 3 μM DHC, and a dose–response test was performed, in which the reaction mixture was exposed in the range of 0-3 μM DHC along with 0 μM, 5 μM, and 10 μM BPZ. Tables S1–S3, indicating the volumes used during the studies, are included to make it easier for other researchers to replicate the results (Supplementary Materials—Reaction conditions for the enzyme activity experiments (Tables S1–S3)).

### 4.3. Apparatus and HPLC-DAD Conditions

Analysis was performed using an LC-20AD Shimadzu (Shimadzu Corporation, Canby, OR, USA) liquid chromatograph. Detection was carried out at a wavelength of 240 nm using a Shimadzu 364 SPD-M20A detector (Shimadzu Corporation, Canby, OR, USA). The DAD detector was set within the 200–600 nm range. All chromatographic measurements were controlled by a CTO-10ASVP thermostat (Shimadzu Corporation, Canby, OR, USA). During studies of the inhibition of BPZ, a product of the conversion of cortisol to corticosterone, and a standard of corticosterone, were injected into the columns using a Rheodyne 20 μL injector. Data acquisition and processing were carried out with LabSolutions software (C191-E018, Shimadzu Corporation, Kyoto, Japan). The analysis was performed on an Acquity UPLC BEH C18 column (50 × 2.1 mm, 1.7 μm) from Waters. The mobile phase consisted of 0.1% formic acid in water (solvent A) and 0.1% formic acid in acetonitrile (solvent B). During HPLC experiments, a gradient elution was used as follows: start with 30% B; 0–2 min, 100% B; and 2–8 min, 100% B; then, to return to the initial concentration of B, 8–9 min, 30% B, followed by the next conditioning of 9–15 min, 30% B. The eluent flow rate was maintained at 0.4 mL/min. To avoid excessive pressure increase, some analyses were performed at a lower mobile phase flow of 0.35 or 0.25 mL/min.

### 4.4. Linearity and the Standard Solutions for the Calibration Curve of Corticosterone

Linearity of the calibration curve was estimated for the peak area of each corticosterone standard. The calibration curve was constructed by analyzing the corticosterone standards at concentrations ranging from 0.1 μM to 577 μM and obtained by means of the least squares method. The graph is presented in Figure S1 as Supplementary Materials.

### 4.5. Limit of Detection (LOD) and Limit of Quantitation (LOQ) of Corticosterone

The limit of detection (LOD) and limit of quantification (LOQ) obtained for corticosterone were calculated according to the formulas LOD = 3.3 (SD/S) and LOQ = 10 (SD/S), where SD is the standard deviation of response (peak area) and S is the slope of the calibration curve. The HPLC analyses of corticosterone standards were repeated three times.

### 4.6. Kinetic Analysis of Inhibitory Effects of Bisphenol Z on 11β-Hydroxysteroid Dehydrogenase 1 (11β-HSD1)

A kinetic study of 11β-HSD1 was performed for bisphenol Z (BPZ), the most potent inhibitor of the enzyme. The mode of inhibition and value of the inhibition constant (Ki) of BPZ were investigated using Lineweaver-Burk plots (Figure 11 and Figure 12). The obtained results showed that BPZ exhibits mixed inhibition behavior. The Ki value was estimated from the replots of the slope of the individual Lineweaver-Burk plots versus the inhibitor concentrations (Figure 10). The intersection of plot lines occurred outside both the x- and y-axes, indicating a mixed-type inhibition of 11β-HSD1 activity (Figure 11). This suggests that BPZ can bind both to the active site and to an allosteric site on the enzyme, thereby only partially influencing the binding of the substrate in the active site. The Ki value for 11β-HSD1 inhibition by BPZ was estimated at 3 μM, based on the secondary replot derived from the Lineweaver-Burk plots. The HPLC analyses of corticosterone as a product of the inhibition of BPZ during the kinetic study were repeated three to eight times.

### 4.7. Molecular Docking

Protein structures were retrieved from the RCSB PDB and AlphaFold DB as follows: human 11β-HSD1 (PDB ID: 3OQ1, 2.60 Å), rat 11β-HSD1 (PDB ID: 5QIJ, 2.65 Å), and Arabidopsis thaliana 11β-HSD2 (AlphaFold predicted model for UniProt Q9STY8). As no experimental structure is available for AtHSD2, we used the AlphaFold-predicted structure, treated it with the same preprocessing pipeline, and reported docking results for AtHSD2 as model-based with appropriate caveats. Protein preprocessing was performed using ChimeraX: all nonstandard residues and water molecules were removed, and for structures containing identical chains, only one chain was retained. The bisphenol Z ligand was obtained from PubChem (https://pubchem.ncbi.nlm.nih.gov/, accessed on 15 July 2025) and geometry-optimized using Avogadro to obtain the lowest-energy conformer.

For docking preparation, AutoDock Tools was used. Polar hydrogens were added, Kollman charges were assigned to proteins, and Gasteiger charges were assigned to the ligand. Both proteins and ligands were then saved in PDBQT format. Grid boxes were defined around the active sites, and docking simulations were performed using the Lamarckian genetic algorithm with the macromolecule set as rigid. All parameters were set to default unless otherwise specified. The resulting docking poses were analyzed and visualized using AutoDock 4.2 Discovery Studio.

## 5. Conclusions

The biological actions of some bisphenols are still relatively unknown and have not been sufficiently explained. To address this data gap, we aimed to evaluate the ability of BPZ to inhibit rat 11-hydroxysteroid dehydrogenase 1 (11β-HSD1) enzyme activity. In conclusion, we found that bisphenol Z is the most potent inhibitor of the rat 11β-HSD1 enzyme. The intersection of plot lines occurred outside both the x- and y-axes on the Lineweaver-Burk plots, indicating a mixed-type inhibition of 11β-HSD1 activity by BPZ. This suggests that BPZ can bind to both the active site and an allosteric site on the enzyme, thereby only partially influencing the binding of the substrate in the active site. The Ki value for 11β-HSD1 inhibition by BPZ was estimated at 3 μM, based on the secondary replot derived from the Lineweaver-Burk plots. By varying the concentration of substrate (11-dehydrocorticosterone (DHC)) for each of the examined concentrations of BPZ as an inhibitor, the resulting saturation curves were compared using an Eadie-Hofstee plot. The results obtained as Eadie-Hofstee plots, in which the saturation curves intersect on the y-axis or between the y- and x-axes, may confirm that a mixed mode of inhibition is indeed operating. Molecular docking data and analysis of the interaction profiles revealed that bisphenol Z consistently occupies the active site cavities of all three enzymes, forming a stabilizing network of non-covalent interactions. Across all complexes, the binding mode is predominantly governed by van der Waals contacts, π-π stacking, and π–alkyl interactions, supplemented by a limited number of conventional hydrogen bonds.

## Figures and Tables

**Table 1:** Comparison of Lineweaver-Burk, Eadie-Hofstee, and Hanes-Woolf plots.

Plot	X-Axis and Y-Axis	Slope and Point of Intersection with Y-Axis	Equation	Comments
Lineweaver-Burk	1/[*S*]	*K_m_*/*V_max_*	$\frac{1}{v}=\frac{K_{m}}{V_{max}}\frac{1}{\left[S\right]}+\frac{1}{V_{max}}$	
				Highlights the errors at low concentrations
	1/*V*	1/*V_max_*		
Eadie-Hofstee	*V*/[*S*]	−*K_m_*	$v=V_{max}-K_{m}\frac{v}{\left[S\right]}$	
	*V*	*V_max_*		The points may merge at extremely high and low concentrations
Hanes-Woolf	[*S*]	1/*V_max_*	$\frac{\left[S\right]}{v}=\frac{1}{V_{max}}\left[S\right]+\frac{K_{m}}{V_{max}}$	
	[*S*]/*V*	*K_m_*/*V_max_*		Better error distribution than Lineweaver-Burk plots

**Table 2:** Binding parameters for bisphenol Z with 11β-HSD isoforms.

Ligand + Protein Combination	Estimated Free Energy of Biding (kcal/mol)	Final Intermolecular Energy (kcal/mol)	vdW + Hbond + Desolv Energy (kcal/mol)	Electrostatic Energy (kcal/mol)	Final Total Internal Energy (kcal/ mol)	Torsional Free Energy (kcal/mol)	Unbound System’s Energy (kcal/mol)	Estimated Inhibition Constant, Ki (nM)
**Bisphenol Z + 11β-HSD1 (human)**	−8.21	−9.41	−9.06	−0.34	−0.99	1.19	−0.99	953.93
**Bisphenol Z + 11β-HSD1 (rat)**	−8.29	−9.48	−9.39	−0.09	−0.91	1.19	0.91	839.63
**Bisphenol Z + 11β-HSD2 (Arabidopsis)**	−8.06	−9.26	−9.22	−0.04	−0.78	1.19	−0.78	1230

**Table 3:** Aromatic/π-π interactions, hydrophobic contacts, and hydrogen bonds for three kinds of enzymes.

Enzyme	Aromatic/ π-π Interactions	Hydrophobic Contacts	Hydrogen Bonds
**Human 11β-HSD1**	Tyr183	Ile218, Leu215, Ala223	Asn119, Lys187
**Rat 11β-HSD1**	Tyr158, Ala198	Ile193	Gly16, Ile193
**Arabidopsis 11β-HSD2**	Phe227, Tyr196, Thr185	–	Gln136, Ser183

## Data Availability

Data are contained within this article and the Supplementary Materials. Authors are willing to share the original data and materials upon request.

## Supplementary Materials

The following supporting information can be downloaded at https://www.mdpi.com/article/10.3390/molecules30193941/s1: Figure S1. Calibration curve of corticosterone in the concentration range of 0.1 μM–577 μM. Table S1. Example preparation of the reaction mixture for the enzyme activity experiments with BPZ at 5 μM. Table S2. Example preparation of the reaction mixture for the enzyme activity experiments with BPZ at 10 μM. Table S3. Example preparation of the reaction mixture for the enzyme activity experiments with BPZ at 20 μM.

## Abbreviations

The following abbreviations are used in this manuscript:

MCBPA	3-chlorobisphenol A
BPZ	Bisphenol Z
TrCBPA	3,3′,5-trichlorobisphenol A
TCBPA	Tetrachlorobisphenol A
TBBPA	Tetrabromobisphenol A
