# Supplementary material for: Inhibitory Effects of Bisphenol Z on 11β-Hydroxysteroid Dehydrogenase 1 and In Silico Molecular Docking Analysis
Source: Molecules. 2025 Oct 1;30(19):3941. doi: 10.3390/molecules30193941 (PMC12525740; doi:10.3390/molecules30193941)
Supplement: Supplementary file 1 [file molecules-30-03941-s001.zip › molecules-3854288-supplementary.pdf]

molecules

MDPI

Article

Inhibitory effects of Bisphenol Z on 11β-hydroxysteroid dehydrogenase 1 and *in silico* molecular docking analysis

Tomasz Tuzimski [1]*, Mateusz Sugajski [2]

[1] Department of Physical Chemistry, Faculty of Pharmacy, Medical University of Lublin, Chodźki 4a, 20-093 Lublin, Poland
[2] Department of Environmental Chemistry and Bioanalytics, Faculty of Chemistry, Nicolaus Copernicus University, Gagarina 7, 87-100 Torun, Poland; mateusz.sugajski@o2.pl
* Correspondence: tomasz.tuzimski@umlub.pl

Supplementary Materials - Linearity, standard solutions for calibration curve of corticosterone

Linearity of the calibration curve was estimated for the peak area of each corticosterone standards. Calibration curve was constructed by analyzing the corticosterone standards at concentrations ranging from 0.1 µM to 577 µM and obtained by means of the least square method. The graph is presented in **Figure S1**.

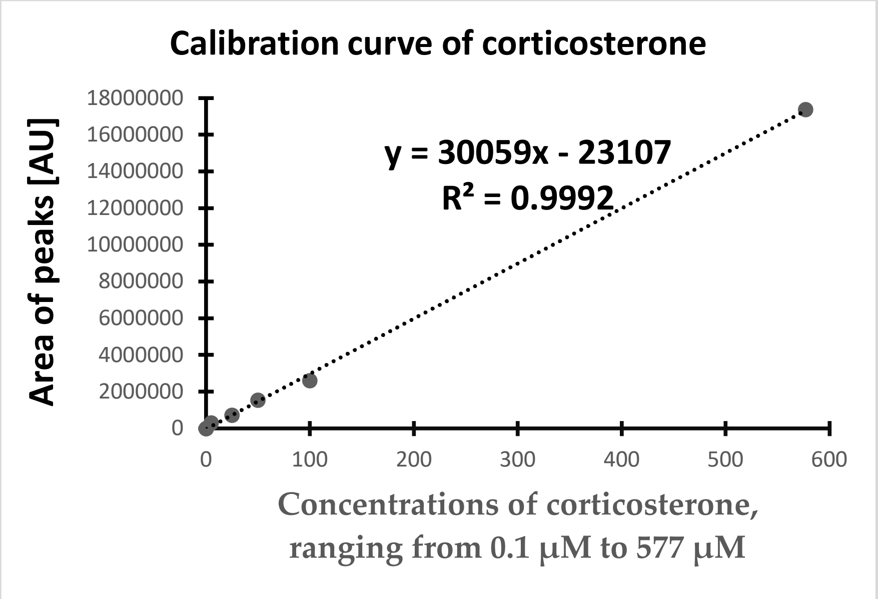

Figure S1. Calibration curve of corticosterone in the concentration range of 0.1 µM–577 µM.

Supplementary Materials—Reaction conditions for the enzyme activity experiments (Tables S1, S2, S3).

Table S1. Example preparation of the reaction mixture for the enzyme activity experi-ments with BPZ at 5 µM.

Concentration of DHC	Buffer BPS	BPZ	D-Glucose 6-phoshate	DHC	NADPH	11β-HSD1

			**sodium salt**			
**1 µM**	216.6 µl	8.4 µl	100 µl	25 µl	100 µl	50 µl
**2 µM**	191.6 µl	8.4 µl	100 µl	50 µl	100 µl	50 µl
**2.5 µM**	179.1 µl	8.4 µl	100 µl	62.5 µl	100 µl	50 µl
**3 µM**	166.6 µl	8.4 µl	100 µl	75 µl	100 µl	50 µl

Table S2. Example preparation of the reaction mixture for the enzyme activity experiments with BPZ at 10 µM.

**Concentration of DHC**	**Buffer BPS**	**BPZ**	**D-Glucose 6-phoshate sodium salt**	**DHC**	**NADPH**	**11β-HSD1**
**1 µM**	208.2 µl	16.8 µl	100 µl	25 µl	100 µl	50 µl
**2 µM**	183.2 µl	16.8 µl	100 µl	50 µl	100 µl	50 µl
**2.5 µM**	170.7 µl	16.8 µl	100 µl	62.5 µl	100 µl	50 µl
**3 µM**	158.2 µl	16.8 µl	100 µl	75 µl	100 µl	50 µl

Table S3. Example preparation of the reaction mixture for the enzyme activity experiments with BPZ at 20 µM.

**Concentration of DHC**	**Buffer BPS**	**BPZ**	**D-Glucose 6-phoshate sodium salt**	**DHC**	**NADPH**	**11β-HSD1**
**1 µM**	191.4 µl	33.6 µl	100 µl	25 µl	100 µl	50 µl
**2 µM**	166.4 µl	33.6 µl	100 µl	50 µl	100 µl	50 µl
**2.5 µM**	153.9 µl	33.6 µl	100 µl	62.5 µl	100 µl	50 µl
**3 µM**	141.4 µl	33.6 µl	100 µl	75 µl	100 µl	50 µl
